# Tachycardia-Induced Cardiomyopathy in an Infant with Atrial Flutter and Prolonged Recovery of Cardiac Function

**DOI:** 10.3390/jcm13113313

**Published:** 2024-06-04

**Authors:** Tomohide Sakai, Kaori Tsuboi, Shinya Takarada, Mako Okabe, Hideyuki Nakaoka, Keijiro Ibuki, Sayaka W. Ozawa, Yukiko Hata, Shojiro Ichimata, Naoki Nishida, Keiichi Hirono

**Affiliations:** 1 Department of Pediatrics, Faculty of Medicine, University of Toyama, Toyama 930-0194, Japan; xte3211tomo@gmail.com (T.S.);; 2 Department of Legal Medicine, Faculty of Medicine, University of Toyama, Toyama 930-0194, Japan

**Keywords:** tachycardia-induced cardiomyopathy, atrial flutter, heart failure

## Abstract

**Background:** Tachycardia-induced cardiomyopathy (TIC) is caused by prolonged tachycardia, leading to left ventricular dilatation and systolic dysfunction with heart failure. Although TIC is more common in adults, it is rare in early infancy. **Methods:** Clinical testing was performed as part of medical evaluation and management. Next-generation sequencing (NGS) was conducted for a patient with TIC. A literature review on TIC was also conducted. **Results:** The case involved a 5-month-old infant referred to the hospital due to symptoms of heart failure lasting at least two months. The infant’s heart rate was 200 beats per minute, the left ventricular ejection fraction fell below 14%, and electrocardiograms showed atrial flutter, suggesting TIC. After cardioversion, there was no recurrence of atrial flutter, and cardiac function improved 98 days after tachycardia arrest. The NGS did not identify any pathogenic variants. The literature review identified eight early infantile cases of TIC. However, no previous reports described a case with such a prolonged duration of TIC as ours. **Conclusions:** This is the first report of a case of prolonged TIC in a child with the documented time to recover normal cardiac function. The improvement of cardiac function depends on the duration of TIC. Early recognition and intervention in TIC are essential to improve outcomes for infantile patients, as timely treatment offers the potential for recovery.

## 1. Introduction

Tachycardia-induced cardiomyopathy (TIC) is a myocardial disorder caused by prolonged tachycardia, leading to left ventricular (LV) dilatation and systolic dysfunction with clinical symptoms of heart failure (HF) and palpitations. The most common arrhythmias associated with TIC are ectopic atrial tachycardia (AT) (59%), permanent junctional reciprocating tachycardia (PJRT) (23%), and ventricular tachycardia (7%) [1]. The occurrence of TIC varies for each arrhythmia, ranging from 10% in patients with AT to 50% in patients with atrial fibrillation [2,3,4]. In adults, atrial fibrillation is the most significant cause of TIC, while in children, AT and PJRT are the more common causes [1,5].

TIC is completely or partially reversible after the normalization of the heart rate (HR) [6,7,8]. In infants, the ventricular rate during tachycardia is much faster than normal, and since they cannot verbalize initial symptoms, signs of cardiac failure often do not appear until several days after the onset of tachycardia [9]. Moreover, compared with elderly TIC patients, newborns and infants with TIC tend to present with objective symptoms, such as dyspnea or poor weight gain, rather than subjective symptoms, leading to prolonged TIC.

However, TIC due to atrial flutter (AFL) is very rare, and there have been no reported cases of infants who are late gadolinium enhancement (LGE)-negative but take considerable time to recover normal cardiac function. Here, we report the case of an infant with TIC due to AFL, negative for LGE, who took a long time to recover normal cardiac function and had no genetic background suggestive of cardiomyopathy. Additionally, a literature review was conducted to explore preventative measures and early detection tactics and to determine the severity and prognosis of TIC in infants.

## 2. Materials and Methods

Written and informed consent was obtained. Clinical testing was performed as part of the medical evaluation and management at Toyama University Hospital. This study’s protocol conformed to the ethical guidelines of the 1975 Declaration of Helsinki, as reflected in the prior approval by the Research Ethics Committee of the University of Toyama in Japan (IRB # I2017003). Written informed consent was obtained from the patient’s parents and brother according to institutional guidelines.

### 2.1. Variant Screening

After obtaining informed consent from the patient’s parents, DNA was isolated from whole blood samples. Next-generation sequencing (NGS) of 203 cardiac disease-related genes associated with cardiomyopathy and channelopathies was performed using the Ion PGM System (Life Technologies, Carlsbad, CA, USA). Only variants present in genes outside the top 1% of highly variable genes and meeting acceptable quality scores (Phred-scaled genotype quality score ≥ 20; read depth ≥ 15; and allele fraction ≥ 35) were included. Following quality control procedures, a total of 303,993 variants were obtained from the dataset. These variants were utilized for downstream analysis. Sanger sequencing was used to validate the results of NGS for all pathogenic variant candidates that passed these selection criteria. For this purpose, the nucleotide sequences of the amplified fragments were directly sequenced bidirectionally using the Big Dye Terminator v3.1 Cycle Sequencing Kit (Applied Biosystems, Foster City, CA, USA), and sequence analysis was conducted using the ABI 3130xl automated sequencer (Applied Biosystems, Waltham, MA, USA).

### 2.2. Data Analysis and Variant Classification

We conducted primary, secondary, and tertiary analyses, including the optimization of signal processing, base calling, sequence alignment, and variant analysis, using Torrent Suite and Ion Reporter Software 5.0 (Life Technologies, Carlsbad, CA, USA). The allele frequencies of all detected variants were determined using the gnomAD (v2.1.1) database, which includes data from 1208 Japanese individuals, and the Japanese Multi Omics Reference Panel (jMorp) ToMMo 54KJPN. Variants with a minor allele frequency of 0.005 or higher in the gnomAD and HGVD populations were excluded. The variant evaluation was manually performed based on detailed information obtained from ClinVar (https://www.ncbi.nlm.nih.gov/clinvar/, accessed on 31 March 2024) and the Human Gene Mutation Database (HGMD, http://www.hgmd.cf.ac.uk/ac/index.php, accessed on 31 March 2024). Variants were classified according to the American College of Medical Genetics and Genomics (ACMG) guidelines. To assess the pathogenicity of the remaining variants, we utilized five different in silico prediction algorithms: SIFT, Align GVGD, MutationTaster2, PolyPhen-2, and CADD. Variants predicted to be deleterious or pathogenic by at least four of the five in silico algorithms were considered to have a high likelihood of pathogenicity.

### 2.3. Literature Review

We searched PubMed (from its inception until 31 March 2024) for publications about TIC in infants using the search phrases “tachycardia-induced cardiomyopathy” and “early infant” [Title/Abstract]. Our search strategy included gene names in the titles and abstracts. We also checked the bibliographies of all relevant studies and reviews identified. Two investigators independently reviewed the articles. During the initial screening, the titles and abstracts of all articles were reviewed, and those that met the exclusion criteria were excluded. In the secondary screening, all remaining articles were reviewed for eligibility. The following variables were extracted from each study: age at TIC onset, symptoms, cause of tachycardia, HR, duration from onset, left ventricular (LV) function in an echocardiogram at initial presentation and after treatment, medication, non-pharmacological therapy, and prognosis. The primary outcome was the prognosis of patients with TIC. The secondary outcomes were the treatment methods and LV function after treatment.

## 3. Results

**Figure 1:**
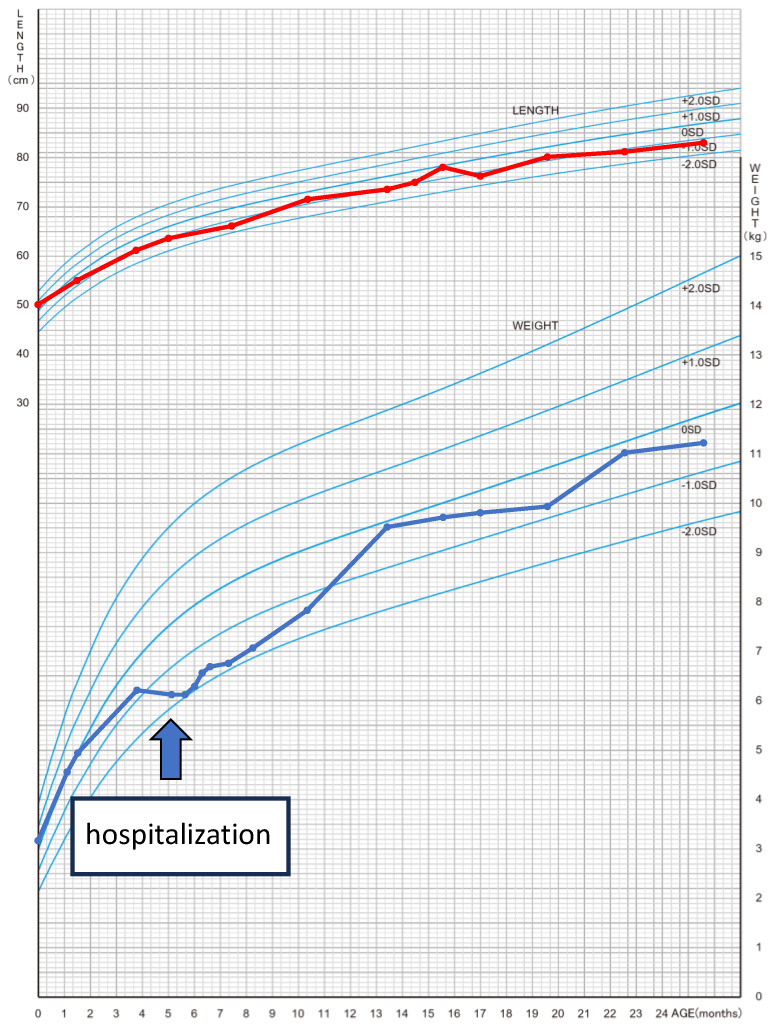
Cross-sectional growth chart showing growth retardation in patients during TIC. Red line shows the growth of body height. Blue line shows the growth of body weight. This table was generated from the formula in reference [10].

The patient was a 5-month-old boy. He was born at 39 weeks and 3 days by normal vaginal delivery, with a birthweight of 3144 g and Apgar scores of eight points at 1 min and nine points at 5 min after an uncomplicated pregnancy. He had no previous or family history of any illness. He had been losing weight for 2 months prior to admission (Figure 1). One month before hospital admission, he developed a wet cough that did not improve with any medication, and wheezing was also observed. Five days before hospital admission, the patient had poor feeding, and the local clinic noted tachycardia with a rate of 190 beats per minute (bpm). Steroids were prescribed for the wheezing, but these symptoms did not improve. On the day of hospital admission, a chest X-ray showed an enlarged cardiothoracic ratio, and an echocardiogram showed reduced LV contraction. The patient was urgently transferred to our hospital.

Upon admission, he was drowsy, with a pale face, cold extremities, and the following vital signs: a pulse of 200 bpm, a blood pressure of 50 mmHg systolic and 24 mmHg diastolic, a percutaneous oxygen saturation (SpO2) of 98% in room air, a respiratory rate of 40 breaths per minute, and a body temperature of 36.3 degrees Celsius. A mild systolic murmur (Levine grade 1/6) was audible at the left sternal border. There were coarse crackles in both lungs. A slightly hard liver was palpated 2 cm below the costal margin. There was no edema in the extremities, but peripheral coldness was present, and the capillary refill time was more than 2 s. There was no family history of cardiomyopathy.

Laboratory tests revealed high levels of NT-proBNP (8320 pg/mL) and troponin I (69.7 pg/mL). The other laboratory data and bloody gas data results were as follows: in the blood tests, the WBC was 12,260/μL (Neut% 73.0%), the Hb level was 9.8 g/dL, the PLT level was 453,000/μL, the AST level was 35 U/L, the ALT level was 15 U/L, the LD level was 311 U/L, the CK level was 29 U/L, the Nt-proBNP level was 8320 pg/mL, the troponin I level was 69.7 pg/mL, the CRP level was 0.17 mg/dL, the TSH level was 2.26 mIU/L, the free T3 level was 2.2 pg/mL, the free T4 level was 1.4 ng/dL, and the H-FABP result was negative; the blood gas (venous blood, after intubation) tests obtained pH 7.500, a pCO2 level of 31.2 mmHg, a HCO_3_^−^ level of 24.1 mmol/L, a BE level of 1.6 mmol/L, and a Lac level of 1.0 mmol/L.

**Figure 2:**
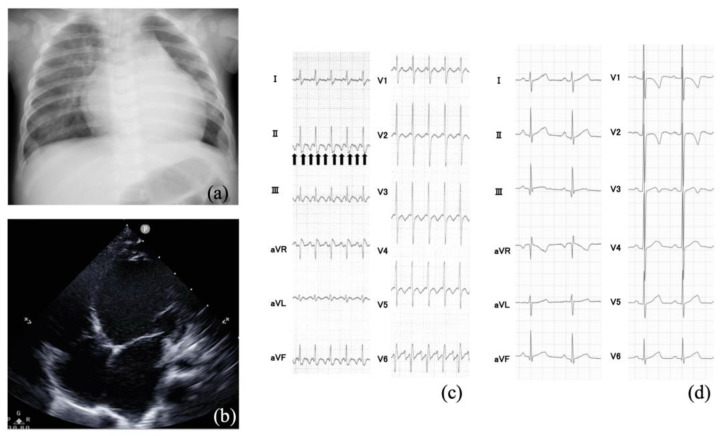
(**a**) Chest X-ray showing cardiomegaly with a cardiothoracic ratio of 0.62 and lung congestion. (**b**) Echocardiography shows that the left ventricular dilation and wall motion are diffusely decreased. (**c**) Typical atrial flutter with 2:1 conduction. The arrows indicate the flutter waves. (**d**) Sinus rhythm after synchronized electrical cardioversion.

The cardiothoracic ratio was 0.63, and lung congestion was observed in the chest X-ray (Figure 2a). The echocardiography showed diffusely decreased LV wall motion (Figure 2b). The LV ejection fraction (LVEF) was 13.7%, and the LV end-diastolic diameter (LVDD) was 39.7 mm (7.0 SD). In the electrocardiogram (ECG), the heart rate was 200 bpm, and a sawtooth wave was observed (Figure 2c).

The patient was determined to be in shock and immediately admitted to the intensive care unit. He was intubated, ventilated, and started on dopamine (5 µg/kg/min), dobutamine (5 µg/kg/min), adrenaline (0.04 µg/kg/min), and olprinone (0.3 µg/kg/min) for acute HF treatment. However, the patient’s heart rate increased to more than 200 bpm, and hypotension persisted. Sawtooth waves were visible in the twelve-lead ECG. We ruled out hereditary cardiomyopathy or secondary cardiomyopathy as the cause of acute HF. Structural heart abnormalities were not detected, and the patient’s mental and motor development were normal. Metabolic or neuromuscular diseases were denied. We suspected TIC due to AFL as the most likely cause. Therefore, synchronized electrical cardioversion was performed (1 J/kg), and a normal sinus rhythm was achieved (Figure 2d). Treatment for HF was continued with dopamine, dobutamine, adrenaline, and olprinone. The LVEF remained at around 20%, and the LVDD was 40.0 mm (7.2 SD). Dopamine was discontinued on the 5th day, and adrenaline was discontinued on the 11th day after admission. Meanwhile, angiotensin-converting enzyme inhibitors and carvedilol were added. Extubation was performed on the 12th day. Tolvaptan was introduced for congestion on the 16th day. Dobutamine was terminated on the 21st day and olprinone on the 29th day. The serum level of NT-proBNP decreased from 8320 pg/mL on day 1 to 129 pg/mL on day 40, and AFL did not recur. He was discharged from the hospital on the 41st day, with an LVEF of 40% and an LVDD of 33.5 mm (4.4 SD).

Ninety-eight days after admission, the LVEF had improved to 59%, and the LVDD was 30 mm (2.6 SD). At 161 days, the LVEF was 62%, and the LVDD was 31 mm (2.4 SD). Cardiac catheterization performed 10 months after admission (at the age of 15 months) showed an LVEF of 75%, a cardiac index (CI) of 3.8 L/min/m^2^, and an LV end-diastolic pressure of 6 mmHg.

Cardiac magnetic resonance imaging was conducted one year after admission, and the delayed contrast LGE was negative. Additionally, genetic testing via NGS with a cardiovascular disease panel was performed to differentiate primary cardiomyopathy, which did not show any known underlying predisposition.

Now, at 3 years old, he remains healthy with normal mental and motor development.

### Literature Review

Our literature review identified eight cases of early infantile TIC (Table 1): three cases with permanent junctional reciprocating tachycardia and supraventricular tachycardia and two cases with AFL. With the exception of one case, all patients exhibited symptoms. However, the duration of TIC was unclear, except for two cases in which TIC lasted for approximately 1 month. Medications and non-pharmacological therapies were administered in six cases. All cases presented with pronounced tachycardia and reduced LV function; however, their outcomes were generally favorable.

## 4. Discussion

The duration of prolonged tachycardia is crucial for the recovery of cardiac function in patients with TIC. In our case, TIC may have manifested at least 2 months before admission, evidenced by body weight loss without any other identifiable causes. It took 98 days from the initiation of treatment for normal cardiac function to be restored. This represents the first reported case of prolonged TIC with the documented time to recover normal cardiac function.

As our literature review suggested, AFL is an uncommon arrhythmia in newborns and infants. Due to its low incidence in this age group, previous reports have been limited in discussing the most efficacious therapy and expected prognosis. Texter et al. reported that infants with AFL who presented over 3 months were statistically more likely to develop congestive HF [18]. They also found that 34% of patients had AFL complicated by congestive HF, recurrence, another arrhythmia, or AFL refractory to conversion to sinus rhythm [18]. Therefore, early recognition of the relationship between the causative arrhythmia and TIC is necessary to provide treatment that improves symptoms and LV functional status [1].

The mechanism underlying the development of cardiomyopathy due to sustained tachycardia is not fully understood, but several theories have been proposed. Possible mechanisms for cardiac dysfunction in TIC include decreased blood flow to the myocardium [19], the depletion of adenosine triphosphate (ATP) [20], and decreased beta-adrenergic receptors [20]. Sustained tachycardia leads to abnormal cellular remodeling and a reduction in the number of microtubules within myocardial cells, which may contribute to myocardial contractile dysfunction [21]. Chronic tachycardia may also result in the depletion of high-energy phosphate, leading to decreased intracellular sarcolemmal sodium/potassium ATPase activity and an altered enzyme distribution affecting calcium handling [22]. Consequently, infants and young children may have heightened intracellular calcium stores. Pathophysiologically, the pathogenesis of TIC involves remodeling processes such as neurohormonal overactivation, the release of natriuretic peptides, the secretion of inflammatory cytokines, ventricular and atrial dilation without hypertrophy, a background of extracellular matrix changes, reduced systolic and diastolic function, and increased filling pressures [23,24]. Additionally, there are abnormalities in excitation–contraction coupling, the intracellular mitochondrial apparatus, calcium processing, and energy production due to oxidative stress [25,26].

The evaluation of a patient with newly developed HF requires a high index of suspicion for underlying tachyarrhythmias. Systolic ventricular dysfunction is often the first manifestation of TIC observed in echocardiography, followed by prolonged dysfunction and subsequent LV dilation [27]. The differential diagnosis of infants presenting with acute HF and having structurally normal hearts is often challenging due to multiple possible etiologies. These may include infectious, familial, metabolic, mitochondrial, toxic, inflammatory, or neuromuscular causes, with most cases being idiopathic. Genetic and metabolic etiologies such as storage disorders, mitochondrial disorders, and carnitine deficiency should also be considered in infants with newly developed cardiomyopathy. The diagnostic challenge in TIC lies in differentiating it from nonischemic dilated cardiomyopathy (DCM) [24]. DCM is defined as a “myocardial disease characterized by LV or biventricular diastolic and systolic dysfunction despite the absence of pressure or volume overload or coronary artery disease sufficient to explain the dysfunction” [28]. Despite being caused by various etiologies and thus having diverse pathologic manifestations, DCM is the second most frequent HF phenotype after ischemic heart disease and is an indication for cardiac transplantation [29]. Patients with TIC typically present with higher heart rates and lower LVEF at diagnosis, with more significant recovery of LVEF after HR normalization [30]. In contrast, patients with DCM tend to have a wider QRS complex, more frequent LGE, and increased rates of rehospitalization during follow-up [31,32]. In our case, genetic testing was conducted to determine the underlying cause of HF, whether it was TIC or DCM. However, no obvious genetic predisposition was identified. Therefore, TIC should be considered in the diagnosis of patients presenting with DCM of uncertain origin, particularly in those who have a history of tachycardias or AFL.

The duration of prolonged tachycardia plays a crucial role in the recovery of cardiac function in TIC. As TIC is a reversible cause of cardiac dysfunction in children, early diagnosis and effective management are essential [33]. In animal models, the normalization of cardiac output, atrial pressure, calcium uptake, and creatinine kinase function has been reported within 2–4 weeks after the cessation of tachycardia. However, some changes, such as LV fibrosis or dilation, may persist for several weeks despite the removal of tachycardia, potentially causing delayed normalization of LVEF [34,35]. In our case, although it is challenging to determine the exact onset of arrhythmias, TIC may have commenced at least two months before admission, indicated by the infant’s body weight loss without any other identifiable causes. According to a report comprising 50 cases of AFL in infants under one year of age, 36 cases (72%) developed AFL within 48 h of birth, and 44 cases (88%) within 2 weeks of delivery [18]. For children with cardiac dysfunction (LVEF < 50%) and LV dilatation (LVDD z-score > 2.0) due to TIC, the median time to recovery from treatment is reported as 51 days for normalizing LVEF and 71 days for normalizing LVDD [1]. Previous studies have indicated that recovery of LV systolic function takes weeks to months, while reverse remodeling may take months to years [1]. In adults, once HR is normalized through medical therapy, electrical cardioversion, or radiofrequency ablation, clinical and functional recovery of the heart typically occurs rapidly, usually within 6 months [23,36,37]. In pediatric cases, the median time to LVEF normalization has been reported as 1.5 months [38]. Additionally, in other pediatric reports, the median time to recovery was 51 days for LVEF and 71 days for LVDD. Heart transplantation was required in two patients (4%) and one patient (1%) died [1].

The predictors of LV systolic function recovery, as determined by multivariate analysis, included younger age, the percentage of standardized tachycardia, the use of mechanical circulatory support, and an increased LVEF. For the normalization of LV size, only a lower baseline LVDD was identified as a predictor [1]. In our case, the normalization of LVEF took 98 days, and the normalization of LVDD took more days, which exceeded the durations reported in the previous literature reviews. Despite the prolonged recovery of LVEF and LVDD observed in our case, these predictors lend support to our findings and those of the cases in our literature review.

Patients with recovered TIC maintain a large LV volume and some degree of negative remodeling [23,39]. This phenomenon may be attributed to the persistence of interstitial fibrosis despite the recovery of myocardial function [40]. Cardiac MRI with LGE has been demonstrated to aid in the assessment of myocardial fibrosis in various cardiomyopathies [41]. In patients with TIC, LGE may play a role in distinguishing TIC from primary cardiomyopathy and in predicting improvements in LV function. The prevalence of LGE in the TIC group was 16%, and notably, LGE was localized in the mid-myocardium [30]. The presence of LGE in patients with TIC carries prognostic implications and can serve as a predictor of increased cardiac events [42]. Indeed, the presence of LGE was identified as an independent predictor of a lack of recovery of LVEF in TIC patients (*p* = 0.004) [30], and it was associated with the absence of LVEF normalization at 6 months [43]. LGE was more prevalent in the TIC group compared with the DCM group. Furthermore, in multivariate analysis, the presence of LGE emerged as an independent predictor of the lack of LVEF recovery [30]. Therefore, in our case, although the recovery from HF took longer than reported in other cases, we were confident that improvement in HF was possible because LGE was absent.

Sixty-nine percent of TIC patients underwent defibrillation or ablation [31]. Treatment strategies such as antiarrhythmic drug therapy or catheter ablation did not have a significant effect on cardiomyopathy recovery. This suggests that the negative inotropic effects of antiarrhythmic drugs do not necessarily impede myocardial recovery, as previously proposed [44]. Additionally, in a previous report of 24 cases in adult patients, 5 patients with recurrent tachycardia experienced a rapid decrease in LVEF [45], highlighting the importance of preventing recurrent arrhythmias in adult TIC patients. In contrast, recurrence is rare in pediatric patients after the cessation of AFL, indicating that prophylactic administration of antiarrhythmic drugs may be unnecessary [18]. In our case, preventative medication was not administered, and AFL has not recurred for more than 3 years.

## 5. Conclusions

In infants with TIC, the inability of patients to recognize palpitations or articulate symptoms often leads to delays in seeking medical attention, resulting in the development of HF. The extent of cardiac function improvement is contingent upon the duration of TIC. Early recognition and intervention in TIC are essential to improve outcomes for pediatric patients, as timely treatment offers the potential for recovery. Therefore, TIC should be considered for a new diagnosis of LV dysfunction or HF when persistent tachycardia is present in infants. Combining cardiac MRI with genetic testing helps us to determine the prognosis of TIC. Further improvements in preventive measures for TIC and early detection tactics need to be made in future studies.

## Figures and Tables

**Table 1:** Literature review summary with tachycardia-induced cardiomyopathy in early infants.

Authors	Age of TIC	Symptom	Cause of Tachycardia	Heart Rate (Beats/min)	Duration from Onset	LV Function in Echocardiogram at Initial Presentation	LV Function in Echocardiogram after Treatment	Medication	Non-Pharmacological Therapy	Prognosis
Van Hare G. F. et al. [11]	5 weeks old	N/A	PJRT	N/A	N/A	LVFS 5%	Improved	Flecainide and sotalol	Catheter ablation	Alive
Sanchez C. et al. [12]	3 months old	Feeding difficulty and tachypnea	PJRT	230	20 days	LVFS 20%	Improved	Digoxin and amiodarone	Catheter ablation	Alive
Schulze O. C. et al. [13]	3 weeks old	Heart failure	PJRT	230	N/A	LVFS 15%	Improved	No	Catheter ablation	Alive
Mares J. C. et al. [9]	1 month old	Emesis and tachypnea	SVT	260	N/A	LVFS 13%	Improved	Flecainide	No	Alive
Jon Felt, et al. [14]	7 weeks old	Respiratory distress	AFL	195	N/A	LVFS 17%	Improved	Amiodarone	Electrical cardioversion	Alive
Gardiner M. et al. [15]	24 days old	Poor feeding, lethargy, and pallor	SVT	270	N/A	LVEF 35%	Improved	Adenosine	No	Alive
Papadopoulou M. et al. [16]	1 month old	Asymptomatic	AFL	216	N/A	LVFS 19–21%; LVEF 42–45%	Improved	No	Electrical cardioversion	Alive
McKenzie K. et al. [17]	3 months old	Grunting, respiratory distress, and poor feeding	SVT	N/A	1 month	LVEF 22%	Improved	Digoxin, beta-blocker, and amiodarone	Catheter ablation	Alive
This case	5 months old	Respiratory distress and poor feeding	AFL	N/A	2 months	LVEF13.7%	Improved	Beta-blocker and angiotensin-converting enzyme inhibitors	No	Alive

PJRT: permanent junctional reciprocating tachycardia, SVT: supraventricular tachycardia, AFL: atrial flutter, LVFS: left ventricular fractional shortening, and LVEF: left ventricular ejection fraction; TIC; tachycardia induced cardiomyopathy; LV; left ventricle; N/A; not applicable.

## Data Availability

The authors confirm that the data supporting the findings of this study are available within this article.
